# Comparing satisfaction and burnout between caseload and standard care midwives: findings from two cross-sectional surveys conducted in Victoria, Australia

**DOI:** 10.1186/s12884-014-0426-7

**Published:** 2014-12-24

**Authors:** Michelle S Newton, Helen L McLachlan, Karen F Willis, Della A Forster

**Affiliations:** School of Nursing and Midwifery, La Trobe University, Bundoora, VIC 3086 Australia; Judith Lumley Centre, La Trobe University, 215 Franklin St, Melbourne, VIC 3000 Australia; Faculty of Health Sciences, Australian Catholic University, 215 Victoria Parade, Fitzroy, VIC 3065 Australia; The Royal Women’s Hospital, Cnr Grattan St & Flemington Rd, Parkville, VIC 3052 Australia

## Abstract

**Background:**

Caseload midwifery reduces childbirth interventions and increases women’s satisfaction with care. It is therefore important to understand the impact of caseload midwifery on midwives working in and alongside the model. While some studies have reported higher satisfaction for caseload compared with standard care midwives, others have suggested a need to explore midwives’ work-life balance as well as potential for stress and burnout. This study explored midwives’ attitudes to their professional role, and also measured burnout in caseload midwives compared to standard care midwives at two sites in Victoria, Australia with newly introduced caseload midwifery models.

**Methods:**

All midwives providing maternity care at the study sites were sent questionnaires at the commencement of the caseload midwifery model and two years later. Data items included the Midwifery Process Questionnaire (MPQ) to examine midwives’ attitude to their professional role, the Copenhagen Burnout Inventory (CBI) to measure burnout, and questions about midwives’ views of caseload work. Data were pooled for the two sites and comparisons made between caseload and standard care midwives. The MPQ and CBI data were summarised as individual and group means.

**Results:**

Twenty caseload midwives (88%) and 130 standard care midwives (41%) responded at baseline and 22 caseload midwives (95%) and 133 standard care midwives (45%) at two years. Caseload and standard care midwives were initially similar across all measures except client-related burnout, which was lower for caseload midwives (12.3 vs 22.4, p = 0.02). After two years, compared to midwives in standard care, caseload midwives had higher mean scores in professional satisfaction (1.08 vs 0.76, p = 0.01), professional support (1.06 vs 0.11, p <0.01) and client interaction (1.4 vs 0.09, p <0.01) and lower scores for personal burnout (35.7 vs 47.7, p < 0.01), work-related burnout (27.3 vs 42.7, p <0.01), and client-related burnout (11.3 vs 21.4, p < 0.01).

**Conclusion:**

Caseload midwifery was associated with lower burnout scores and higher professional satisfaction. Further research should focus on understanding the key features of the caseload model that are related to these outcomes to help build a picture of what is required to ensure the long-term sustainability of the model.

## Background

Caseload midwifery (also known as one-to-one midwifery, or Midwifery Group Practice (MGP)) offers benefits for women and infants, including a reduction in childbirth interventions [1-3], improved neonatal outcomes [1,2] and greater maternal satisfaction [2,4] compared with standard models of care. Despite the evidence of benefit for women and infants, there is debate about the impact of caseload midwifery on midwives: an important issue in terms of the sustainability and expansion of the model.

Increased professional fulfilment and satisfaction have been reported for midwives working in caseload midwifery [5-9]. Reasons for the high levels of satisfaction include the provision of continuity of care [5], forming relationships with women [8,10], occupational autonomy [5,11-14], personal investment [8], and making a difference to women [15]. However, there are also concerns raised about aspects of caseload midwifery that may have a negative impact on midwives, with discussion of excessive workloads [10,16], long hours spent in on-call work [5,10,14,17], professional isolation [10,18], and difficulty in achieving work-life balance [14,17,19]. While a number of authors have discussed the possibility of burnout in the context of caseload midwifery [19,20], these concerns have not been substantiated in studies where burnout was measured [12,14,21].

Sandall argues that burnout is more likely in the presence of lack of peer and personal support, when midwives work in large groups, and where there are fragmented relationships with women, high workloads and a lack of occupational autonomy [12,13,21]. Key features that have been associated with sustainable caseload midwifery models are thus likely to include occupational autonomy [12,14,21,22], regular time off work [12,22], responsibility for clinical decision-making [21], the availability of social support [12,14,22], job satisfaction and developing relationships with women and their families [12,14,22].

In Australia, there is an increasingly ageing and part-time midwifery workforce, and concerns regarding national and international midwifery shortages [23,24]. Given these factors, it is important that the impact of caseload midwifery models on the workforce is explored; new ways of working for midwives could be linked to recruitment and retention, or on the other hand, to decisions to leave the workplace and/or workforce. There is currently a lack of clear evidence on the impact of caseload midwifery on the workforce, and there needs to be a careful and systematic evaluation of the implementation of new models of midwifery care in terms of the impact on midwives in both the short and long term, and the impact on organisations introducing these models [5,13,25-27].

### Context of this study

In 2007/2008 caseload midwifery was introduced at two public hospitals in Victoria, Australia (two thirds of all births in Australia take place in the public health care system [28]). The Royal Women’s Hospital (the Women’s), a tertiary facility located in Parkville, Melbourne and the place of birth for more than 7000 babies annually, introduced caseload midwifery in the context of a randomised controlled trial (RCT) comparing outcomes for 2,314 low-risk women randomised to receive either caseload midwifery or standard care [29]. Barwon Health, (a regional health service located in Geelong, one hour south-west of Melbourne, and the site of over 2000 births annually), implemented MGP as one of a number of changes to maternity care within the organisation [30].

Within the model at both study sites, the primary midwife provided antenatal care, worked on-call to enable attendance at the woman’s labour and birth, and attended the woman for in-hospital and home-based postnatal care, before handing care over to the community-based Maternal and Child Health (MCH) service, a universal primary care service for families with children from birth to school age [31]. Caseload midwives cared for 40 to 45 women per year (pro-rata for part-time), and provided back-up for their caseload colleagues. The structure and function of these two models was consistent with descriptions of other Australian caseload midwifery models [18,32-35]. The ‘standard’ model of care at both sites was either hospital-based pregnancy, labour, birth and postnatal care provided by hospital midwives and doctors with little continuity of carer (the most common ‘standard care’ option), or a second model known as ‘shared’ care, where pregnancy care is undertaken by a woman’s local doctor (general practitioner), and labour, birth and postnatal care are provided by the hospital midwives and doctors. In the standard care model midwives may work in one practice area (e.g. postnatal ward), or rotate between a number of areas in maternity services, and usually work a shift-based roster.

Prior to the introduction of continuity midwifery models in Victoria, health services are required to negotiate an agreement with the Australian Nursing and Midwifery Federation (ANMF), the national trade union representing nurses and midwives [36]. These negotiations are based on the Victorian Government ‘Midwifery Continuity of Care Models Industrial Framework Agreement’ [37]. The two organisations in this study were required to reach agreement on work conditions for midwives (for those in caseload, and midwives working alongside the model), remuneration arrangements for caseload midwives (to take into account the different way of working, particularly the on call component of the work), and the impact of the introduction of the caseload model on midwifery staffing across the maternity service. According to the industrial agreement that was current at the time of this study, each caseload midwife was required to have a minimum of four clear days off each fortnight (regardless of their full-time or part-time employment status), and could work no more than 12 hours in any 24 hour period [37].

The aim of this study was to compare midwives’ attitudes to their professional role and measures of burnout between caseload midwives and those working in standard care models in these two newly introduced caseload models in Victoria, Australia.

## Methods

### Design

Quantitative data were collected using two cross-sectional surveys; one administered at the commencement of the caseload midwifery model, and the other after the model had been operating for two years. The questionnaires were designed specifically for the study, and included a combination of questions about midwives’ views and experiences of caseload midwifery (these data will be reported separately), two validated scales (one exploring burnout and one exploring midwives’ attitude to their role) and demographic questions (age, years of experience, years in current employment, education, practice area and hours of employment). Open-ended questions were included to provide respondents the opportunity to add free-text responses about positive and negative aspects of caseload midwifery models for midwives generally and for the caseload midwives personally. The responses to these open-ended questions are included in this paper to help interpret the validated scale results. Other open-ended questions focusing on midwives’ work intentions and positives and negatives of the model for women will be reported separately.

The Midwifery Process Questionnaire (MPQ) [9] measures midwives’ attitudes towards their professional role, and focuses on four aspects: professional satisfaction, professional support, client interaction, and professional development [9]. The tool uses five-point Likert-type scales ranging from ‘strongly agree’ (1) to ‘strongly disagree’ (5) with half of the items negatively worded to reduce response bias [38]. Content validity of the tool was assessed by Turnbull and colleagues using a modified Q-sort procedure to ensure that the items related to the four themes [9].

The Copenhagen Burnout Inventory (CBI) [39] measures burnout in three domains; personal burnout, work-related burnout and client-related burnout [39]. The nineteen item tool uses a five-point Likert-type scale, with twelve of the questions using response categories ‘Always’, ‘Often’, ‘Sometimes’, ‘Seldom’, ‘Never/almost never’, and the remaining seven items use response categories ‘To a very high degree’, To a high degree’, ‘Somewhat’, ‘To a low degree’, and ‘To a very low degree’. Reliability of the tool was assessed by the original authors, reporting Cronbach’s alpha between items of 0.87 in the personal and work related sub-scales, and 0.85 for the client related burnout scale [40], indicating that items within the sub-scales were well correlated [41].

Piloting and re-piloting of the survey was undertaken prior to commencement of data collection, firstly with members of the research team, and then with six midwives who were not eligible for inclusion in the study. Minor changes to the wording of the non-scale questions were made following piloting.

### Participants

Participants were permanent full-time or part-time midwives working in midwifery roles in either caseload or standard care models. Midwives who were employed on a casual (non-permanent) basis were excluded from the study as it was not possible to determine which clinical area they were working in and how much work they had been undertaking in maternity services.

### Data collection

The baseline survey was distributed at the Women’s in January 2008, and at Barwon Health in July 2008. The two year survey was distributed in December 2009 at the Women’s and in June 2010 at Barwon Health. Reminders were sent to participants two and four weeks after the initial survey distribution. An incentive (an entry to a draw to win movie tickets) was used at both sites to encourage survey returns. For midwives in standard care models, distribution of the survey was through the internal hospital mail system attached to payslips, and return of the anonymous survey by pre-paid envelope was considered consent. Caseload midwives’ participation was sought by written consent, so that their surveys could be linked to a unique identifier for comparison at baseline and two years and to enable linking with in-depth data obtained through face to face interviews. The surveys for this group were sent to a postal address nominated by the participant and also included pre-paid envelopes for survey return.

### Data analysis

Quantitative data were entered into an Access database [42], and imported into STATA Version 10 [43] for analysis. Data cleaning included range and logic checks as well as checks for duplicate records. Data were pooled for the two sites, summarised using descriptive and inferential statistics, and comparisons were made between caseload and standard care midwives. For normally distributed continuous variables, means were compared using t-tests; the Mann–Whitney test was used for comparison of medians otherwise. Chi square and Fisher’s exact were used for comparison of categorical data, testing for equality of percentages in subgroups [44,45].

The CBI and MPQ questions were both re-coded according to the authors’ instructions [46,47]. Scoring of the MPQ required the reversal of the negatively scored items, and items then individually scored from two (for all responses = 1) to minus two (for all responses = 5) to produce a mean score for each respondent in each sub-scale. Using pooled individual scores, group mean scores for caseload and standard care midwives were calculated for each of the sub-scales, ranging from two, representing a very positive attitude, to minus two, representing very negative attitudes [47]. The CBI responses were assigned a value between zero and 100 for each item within the sub-scales, with a mean score for each sub-scale calculated for each individual respondent, and group mean scores calculated for caseload and standard care midwives from the pooling of these individual scores. A score of 50 in any sub-scale indicates a high degree of burnout within that domain [46].

Open-ended responses from surveys were analysed using content analysis. Responses to each of the open-ended questions were examined to identify themes that would group comments into a sub-set, and each comment was then assigned to a relevant sub-set and reported in frequency of responses [48]. Agreement of allocation of comments within a code structure was confirmed with another member of the research team. The frequency of comments relating to a particular theme are reported [48,49].

Ethics approval for this project was provided by the Human Research Ethics Committees of La Trobe University (Approval No. 07–137), the Royal Women’s Hospital (Project 07/01) and Barwon Health (Project 8–16).

## Results

### Participants

**Figure 1:**
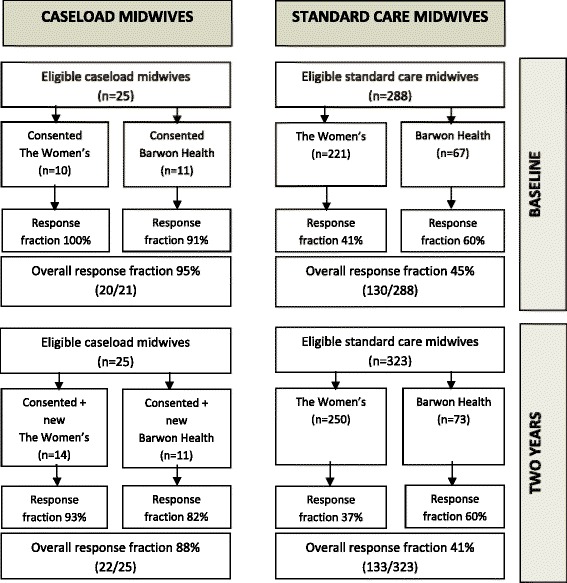
**Survey recruitment and response fractions.**

When caseload midwifery commenced at the study sites, a total of 25 midwives were employed in the model; 21 of these consented to participate in this study. Fourteen of the original consented caseload midwives were still working in the model after two years, and 11 new midwives had joined caseload, making a total of 25 caseload midwives eligible for the two year survey. Of the original caseload midwives, two were on maternity leave at the time of the two year survey and another did not return the second survey despite the reminder cycle. An additional four of the consented caseload midwives resigned from the model in the two and a half year study period and were sent a survey after their resignation. All midwives working in standard care models at both hospitals were also sent two surveys; 288 at baseline and 323 at two years. Response fractions at baseline were 95% for caseload midwives (20/ 21) and 45% (130/288) for midwives in standard care models, and at two years, 88% for caseload (22/25) and 41% (133/323) for midwives in standard care (Figure 1).

Caseload and standard care midwives had similar characteristics in all measures at baseline (Table 1). At two years, caseload and standard care midwives were similar in age, midwifery education setting, and tertiary qualifications, but caseload midwives had fewer years of midwifery experience (p < 0.01) and were more likely to work full-time (p < 0.01) than their standard care counterparts. The percentage of midwives working part-time was very similar to that reported at baseline (65%), and there was also similar representation of shift workers in both surveys (71%).

**Table 1:** **Characteristics of survey respondents by group (caseload and standard care)**

	**Baseline**				**Two years**			
**Characteristics**	***Caseload*** **(n = 21)**		***Standard care*** **(n = 130)**		***Caseload*** **(n = 22)**		**Standard care** ***(n = 133)***	
	**n**	***%***	**n**	***%***	**n**	***%***	**n**	**%**
**Age group**	(n = 20)		(n = 129)		(n = 22)		*(n = 132)*	
20-29 years	2	*10*	22	*17*	7	*32*	24	18
30-39 years	8	*40*	31	*24*	5	*23*	23	17
40-49 years	7	*35*	44	*34*	8	*36*	45	34
>50 years	3	*13*	32	*25*	2	*9*	40	30
**Years in midwifery**	(n = 20)		(n = 129)		(n = 22)		*(n = 131)*	
<1 year	0	*0*	11	*9*	0	*0*	11	8
1-5 years	6	*30*	26	*20*	6	*27*	24	18
6-10 years	5	*25*	26	*20*	8	*36*	10	8
11-15 years	2	*10*	9	*7*	2	*9*	14	11
>15 years	7	*35*	57	*44*	6	*27*	72	55
**Midwifery education**	(n = 20)		(n = 128)		(n = 22)		*(n = 130)*	
Hospital program	8	*40*	61	*48*	7	*32*	70	54
College/university	12	*60*	67	*52*	15	*68*	60	46
Tertiary qualifications*	(n = 20)		(n = 130)		(n = 22)		*(n = 131)*	
Diploma	1	*5*	27	*21*	2	*9*	24	18
Degree	13	*65*	76	*58*	11	*50*	69	53
Post graduate diploma	10	*50*	60	*46*	9	*41*	41	31
Masters degree	0	*0*	13	*10*	2	*9*	11	8
PhD	1	*5*	0	*0*	1	*5*	1	1
None	1	*5*	13	*10*	1	*5*	14	11
**Work hours**	(n = 20)		(n = 129)		(n = 22)		*(n = 131)*	
Full time	7	*35*	49	*38*	14	*64*	44	34
Part time	*13*	65	*80*	62	*8*	36	*87*	66

*Respondents able to select all that applied.

### Comparison of caseload and standard care midwives’ attitudes to their professional role using the Midwifery Process Questionnaire

One hundred and forty eight midwives completed the MPQ questions at baseline (20 caseload and 128 standard care midwives), and 154 midwives at two years (22 caseload and 132 standard care midwives). There were no differences between caseload and standard care midwives in group mean scores for all four sub-scales at baseline (Table 2). After two years, caseload midwives had higher mean scores in the subscales of professional satisfaction, professional support and client interaction compared to midwives in standard care.

**Table 2:** **Comparison of Midwifery Process Questionnaire group mean scores between caseload and standard care midwives**

		**Caseload**			**Standard care**					
	***Survey***	***n***	**Mean score**	**sd**	***n***	**Mean score**	**sd**	**Mean diff**	p* value	**95% confidence interval**
**Professional satisfaction**	*Baseline*	*20*	*0.58*	0.89	*128*	*0.60*	0.67	−0.24	0.89	−0.36, 0.31
	*Two years*	*22*	1.08	0.51	*132*	0.76	0.56	0.32	0.01	0.07, 0.57
**Professional support**	*Baseline*	*20*	0.21	1.09	*128*	0.04	0.68	0.17	0.34	−0.18, 0.52
	*Two years*	*22*	1.06	0.41	*132*	0.11	0.58	0.94	<0.01	0.69, 1.20
**Client interaction**	*Baseline*	*20*	0.1	1.21	*128*	−0.1	0.83	0.2	0.34	−0.22, 0.63
	*Two years*	*22*	1.4	0.38	*132*	0.09	0.71	1.31	<0.01	1.01, 1.61
**Professional development**	*Baseline*	*20*	0.69	0.81	*128*	0.59	0.82	0.1	0.61	−0.29, 0.49
	*Two years*	*22*	0.76	0.73	*132*	0.78	0.65	−0.02	0.9	−0.32, 0.28

*p value calculated using t-test to compare the group mean scores between caseload and standard care.

### Midwifery Process Questionnaire group changes between baseline and two years

**Figure 2:**
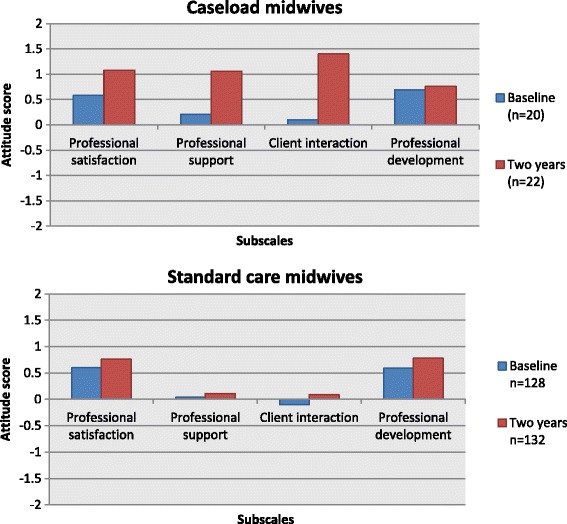
**Midwifery Process Questionnaire scores at baseline and two years for caseload and standard care midwives.**

To explore changes in midwives’ attitudes to their role between baseline and two years, separate analyses were undertaken for caseload and standard care midwives. Caseload midwives scores indicated improvement in mean group scores for professional satisfaction (0.58 to 1.08, p = 0.03, 95% CI −0.95, −0.06); professional support (0.21 to 1.06, p = 0.002, 95% CI −1.35, −0.34) and client interaction (0.1 to 1.4, p < 0.001, 95% CI −1.8, −0.75) between the two surveys, but indicated no change for the professional development subscale (Figure 2). Similar comparisons between surveys were conducted for midwives in standard care, with an improvement in the mean group scores for professional satisfaction (0.6 to 0.75, p = 0.04, 95%, 95% CI −0.31, −0.01); client interaction (−0.1 to 0.09. p = 0.05, 95% CI −0.37, −0.01); and professional development (0.59 to 0.78, p = 0.03, 95% CI −0.38, −0.01), however the strength of the difference was substantially less than that observed in the caseload midwives. No difference was detected in the professional support subscale.

### Midwifery Process Questionnaire individual changes between baseline and two years for caseload midwives

**Figure 3:**
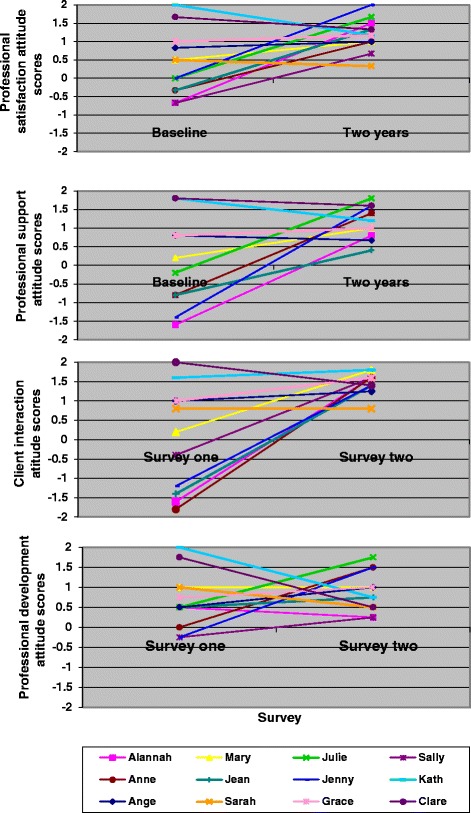
**Midwifery Process Questionnaire changes between baseline and two years for caseload midwives.**

The individual scores of 12 caseload midwives who had answered the MPQ questions at both baseline and at two years were also analysed to determine individual changes over time and the changes in their individual scores for each subscale (Figure 3). There was an increase in the professional satisfaction scores over two years when examining mean group score for these 12 midwives; 0.38 (sd 0.87, range −0.67 to 1.67) compared to 1.18 (sd 0.44, range 0.33 to 2), giving a positive mean difference between surveys of 0.81 (p = 0.02, 95% CI −1.45, −0.17).

A similar pattern was observed in the professional support subscale; there was strong evidence of improved mean group scores for the caseload midwives who responded to both surveys; from 0.05 (sd 1.16, range −1.16 to 1.8) to 1.15 (sd 0.42, range 0.67 to 1.8), mean difference of 1.11 (p = 0.009 95% CI −1.88, −0.33).

There was also strong evidence of improvement in the 12 caseload midwives’ views on their client interaction over two years with the mean group score increasing from −0.02 (sd 1.16, range −1.16 to 1.6) to 1.49 (sd 0.27, range 0.8 to 1.8), with a mean difference of 1.50 (p = 0.003, 95% CI −2.37, −63). There was no change over time in the professional development scores for this sub-sample of 12 caseload midwives.

### Comparison of caseload and standard care midwives’ burnout scores using the Copenhagen Burnout Inventory

One hundred and forty eight midwives responded to the CBI questions at baseline (20 caseload and 128 standard care), and 152 midwives at two years (21 caseload and 131 standard care midwives). At baseline the burnout scores for caseload midwives and standard care midwives were similar for personal burnout (caseload 44.2%; standard care 50.1%, p = 0.17), and work-related burnout (caseload 41%; standard care 45%, p = 0.38). The group mean score for standard care midwives in the personal burnout subscale at baseline was calculated at 50, indicating that as a group, midwives working in standard care were close to burnout according to the CBI. Midwives commencing in the caseload model scored significantly lower than standard care midwives in the client-related burnout subscale at baseline, although both group mean scores were well under the score of 50, indicating that neither group was experiencing burnout associated with the client-related aspects of their work. After two years, the group mean scores for caseload midwives were significantly lower than standard care midwives across all three burnout subscales (Table 3).

**Table 3:** **Comparison of Copenhagen Burnout Inventory group mean scores between caseload and standard care midwives**

		**Caseload**			**Standard care**					
	**Survey**	***n***	**Mean score**	**sd**	***n***	**Mean score**	**sd**	**Mean diff**	p* value	**95% confidence interval**
**Personal burnout**	*Baseline*	*20*	44.2	21.2	*128*	50.1	17.5	−5.9	0.17	−14.5, 2.6
	*Two years*	*21*	35.7	14.0	*131*	47.7	15.6	−12.0	<0.01	−19.2, −4.9
**Work-related burnout**	*Baseline*	*20*	41.1	21.6	*128*	45.1	18.5	−4.0	0.38	−13.0, 5.0
	*Two years*	*21*	27.3	12.4	*131*	42.7	16.2	−15.4	<0.01	−22.7, −8.0
**Client-related burnout**	*Baseline*	*20*	12.3	9.6	*128*	22.4	18.0	−10.1	0.02	−18.2, −2.0
	*Two years*	*21*	11.3	11.9	*131*	21.4	14.9	−10.1	<0.01	−16.9, −3.3

*p value calculated using t-test to compare the group mean scores between caseload and standard care.

### Copenhagen Burnout Inventory group changes between baseline and two years

**Figure 4:**
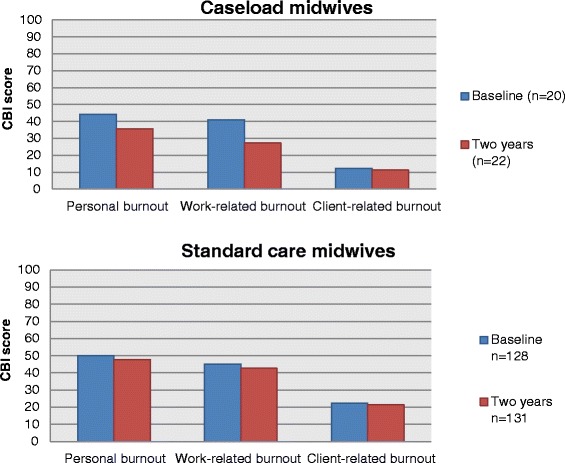
**Copenhagen Burnout Inventory scores at baseline and two years for caseload and standard care midwives.**

Comparisons between the baseline and two year surveys for the caseload and standard care midwives enabled a measure of changes in midwives’ burnout scores over time. Caseload midwives scored significantly lower in the work-related burnout subscale at two years compared to their baseline score (mean difference 13.7, p = 0.02, 95% CI 2.71, 24.84) (Figure 4). There were no differences in the other burnout domains. Similar comparisons of burnout scores for midwives in standard care demonstrated no differences over time.

### Proportion of caseload and standard care midwives classified as ‘burnt out’

The CBI was also used to compare the proportion of midwives in each group who were burnt-out (i.e. had scores ≥ 50) (Table 4). At baseline, a lower proportion of caseload midwives experienced burnout in the personal burnout subscale, however there were no differences in the proportion of midwives burnt-out in the work-related or client-related burnout subscales. After two years there was strong evidence that a lower proportion of caseload midwives were experiencing personal burnout (14% compared to 49%, p < 0.01) and work-related burnout (5% compared to 40%, p < 0.01) compared to those in standard care. Overall, both groups had fewer midwives experiencing burnout in all sub-scales.

**Table 4:** **Percentage of midwives in caseload and standard care identified as ‘burnt-out’ (i.e. scores ≥50), by sub-scale**

	***Baseline***					***Two years***				
	**Caseload (n = 20)**		**Standard Care (n = 128)**		p * value	**Caseload (n = 21)**		**Standard care (n = 131)**		p* value
	n	*%*	n	*%*		n	*%*	n	*%*	
**Personal burnout**	7	*35*	76	*59*	0.05	5	*14*	64	*49*	<0.01
**Work-related burnout**	7	*35*	59	*46*	0.38	2	*5*	52	*40*	<0.01
**Client-related burnout**	0	*0*	10	*8*	0.20	1	*5*	8	*5*	0.89

*p value calculated using chi2.

The CBI scores for all respondents were analysed to see if there was any relationship between age, years of experience, and full-time or part-time work status and burnout scores above 50. At baseline, standard care midwives who were less than 40 years of age were more likely to have scored above 50 on the work-related (57% versus 38%, p = 0.04) and client-related (15% versus 3%, p = 0.02) burnout scales. For caseload midwives, those with less than 10 years’ experience were also more likely to be burnt out on the work-related subscale compared to the caseload midwives with more than 10 years’ experience (55% versus 11%, p = 0.04). At two years, standard care midwives who were less than 40 years of age or had less than 10 years’ experience were still more likely to have work-related burnout than those over 40 years of age or with more than 10 years’ experience (51% versus 32%, p = 0.03 for both comparisons). There was no association between full-time/part-time status and burnout scores.

### Copenhagen Burnout Inventory individual changes between baseline and two years for caseload midwives

**Figure 5:**
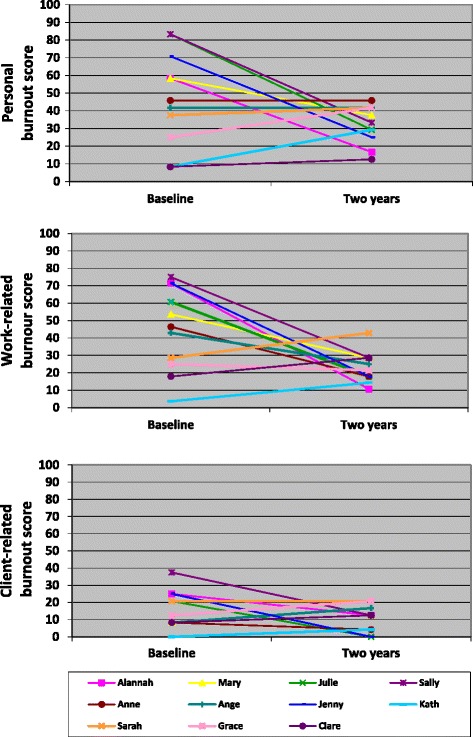
**Copenhagen Burnout Inventory changes between baseline and two years for caseload midwives.**

Eleven caseload midwives completed the CBI questions at both baseline and two years, and the changes in their individual scores for each subscale are represented in Figure 5. Five of the consented caseload midwives who completed both surveys scored greater than 50 in the personal burnout subscale at baseline, but after two years working in the model, all caseload midwives scored less than 50, suggesting that none were experiencing personal burnout. Overall, when the scores of these 11 caseload midwives were combined, there was weak evidence of a decrease in the personal burnout scores (mean group score at baseline 47.4 (sd 26.6, range 8.3 to 83.3) compared to two years 31.1 (sd 10.4, range 12.5 to 45.8), mean difference 16; (p = 0.07, 95% CI −1.8, 34.4).

Similar patterns were seen in the work-related burnout scale. Five of the caseload midwives scored above 50 in the work-related subscale in the baseline survey, indicating that they were experiencing burnout in this domain, but after two years, all 11 caseload midwives scored less than 50. There was strong evidence of a decrease in the mean difference over time for these 11 midwives from 45.1 (sd 24, range 3.6 to 75) to 23.1 (sd 8.9, range 10.7 to 42.9), giving a mean difference of 22.1 (p = 0.02, 95% CI 3.8, 40.3).

No caseload midwives in either survey scored above 50 in the client-related burnout measure. The range of scores at baseline was zero to 37.5 (mean 15.9, sd 10.8) and after two years, zero to 20.8 (mean 10.6, sd 7.5). There was no evidence to suggest a significant change over time for these 11 midwives in their client-related burnout scores (mean difference 5.3, p value 0.21, 95% CI −3.56, 14.16).

### Positive and negative aspects of caseload work for midwives

To further explore midwives’ views and experiences of caseload midwifery and help interpret the results of the measures of attitude to professional role and burnout, caseload and standard care midwives were asked to identify the positive and negative aspects of caseload midwifery for *midwives* in open-ended questions.

#### Positive aspects

Twenty caseload and 117 standard care midwives made one or more comments on the positive aspects of caseload midwifery for midwives at baseline, and 22 caseload and 120 standard care midwives made comments after two years. The themes identified are presented in Table 5. In both surveys the opportunity to work in a model that offers *continuity and relationships with known women* was identified as the most common positive aspect of caseload midwifery for midwives overall (58% at baseline and 65% at two years), followed by *job satisfaction* (41% at baseline and 36% at two years). *Autonomy, responsibility, accountability*, *flexibility of work hours,* and *using midwifery skills and knowledge across the continuum* made up the top five themes at baseline, and after two years *working with women who were more empowered and informed* was also identified by respondents as a positive aspect of caseload midwifery for midwives.

**Table 5:** **Positive aspects of caseload for** ***midwives***

***Theme***	***Baseline caseload (n = 20)***		***Baseline standard care (n = 117)***		***Total baseline (n = 137)***		***Two years caseload (n = 22)***		***Two years standard care*** **(n = 120)**		**Total two years (n = 144)**	
	**n**	***%***	**n**	***%***	**n**	***%***	**n**	***%***	**n**	***%***	**n**	**%**
Continuity and relationships with known women	11	55	68	58	79	58	17	77	76	63	93	65
Job satisfaction	5	25	51	44	56	41	16	73	36	30	52	36
Autonomy, responsibility, accountability	11	55	36	31	47	34	5	23	21	18	26	18
Flexibility (work hours, no shifts)	14	70	28	24	42	31	12	55	23	19	35	24
Utilising midwifery skills and knowledge across all practice areas	8	40	27	23	35	26	2	9	17	14	19	13
Caring for women who are more empowered and informed	5	25	12	10	17	12	5	23	34	28	39	27
Women-centred, holistic	2	10	9	7	11	8	2	9	6	5	8	6
Teamwork	3	15	7	6	10	7	2	9	4	3	6	4
Raising profile of midwifery	1	5	7	6	8	6	3	14	3	2	6	4
Increased self-confidence	1	5	3	3	4	3	-	*-*	1	1	1	1
Lighter workload/more controlled/‘normal’	-	-	4	3	4	3	-	*-*	2		2	1
Improved lifestyle (more time at home)	1	*5*	1	1	4	3	6	22	1	1	7	5
Good remuneration	-	-	1	1	1	1	-	*-*	2	2	2	1
Assists with ward/unit workload	*-*	*-*	*1*	1	1	1	*-*	-	4	3	4	3

Responses to this question were similar between caseload and standard care midwives, although there were slight differences in perceptions of the model at baseline, where the most frequent positive aspect identified by caseload midwives was *flexibility*, whereas the responses from the standard care midwives’ was *continuity and relationships with known women*. However, after two years the three most frequent responses were the same for both groups; *continuity and relationships with known women, job satisfaction* and *flexibility.*

For caseload midwives the frequency that *job satisfaction* was mentioned as a positive increased from 25% (5/20) to 73% (16/22), as did *improved lifestyle* (from 5% (1/20) to 22% (6/22)), while the frequency that caseload midwives mentioned *using midwifery knowledge and skills across the continuum decreased* from 40% (8/20) to 9% (2/22), as did *flexibility* from 70% (14/40) to 55% (12/22).

In addition to the views on caseload for midwives, 22 caseload midwives responded to a question about aspects of the model that they found positive for them personally (Table 6). When the question about positive aspects was phrased to explore the caseload midwives’ personal experience, *flexibility* of the model in terms of work hours and not working shifts (particularly night shift) was mentioned most frequently (15/22), followed by *job satisfaction* (14/22) and i*mproved lifestyle* (8/22). These three aspects could be considered personal benefits, i.e. they are not about the model outcomes, but reflect how these midwives feel about the style of work that they are engaged in.

**Table 6:** **Positive aspects of caseload identified by caseload midwives for themselves personally (two year survey only)**

***Theme***	***(n = 22)***
Flexibility (work hours, no shifts)	*15*
Job satisfaction	*14*
Continuity and relationships with known women	*12*
Improved lifestyle (more time at home, more sleep)	*8*
Improved outcomes (individualised care, quality, more information, confidence)	*7*
Team work (collaboration)	*6*
Autonomy	*5*
Utilising midwifery skills and knowledge	*2*
Good remuneration	*1*
Being pioneers (establishing the model)	*1*
Supportive hospital	*1*

#### Negative aspects

Comments regarding negative aspects of the caseload model for *midwives* were made by 16 caseload and 110 standard care midwives at baseline, and 20 caseload and 117 standard care midwives at two years. The themes identified are presented in Table 7. *On-call work* was the most frequently listed negative aspect of the caseload model, identified by both caseload and standard care midwives in both surveys. Nearly every caseload midwife listed this as a negative feature of caseload midwifery, with comments reflecting that on-call was *unpredictable* and *uncertain*. Equally, the midwives in standard care mentioned on-call work more frequently than any other response. At baseline, *the demands of the role* were identified by 38% of caseload midwives, but after two years this issue was made by only one midwife. *Working long hours* was the third most frequent response in the baseline survey (25%), and the second most common after two years (20%). The remainder of the responses in both surveys were diverse; there were 11 factors identified by caseload midwives as negative aspects of caseload midwifery for midwives, but many of these were mentioned by only one or two respondents.

**Table 7:** **Midwives’ views of the negative aspects of caseload for midwives**

***Theme***	***Baseline caseload (n = 16)***		***Baseline standard care (n = 110)***		***Total baseline (n = 126)***		***Two year caseload (n = 20)***		***Two year standard care (n = 117)***		**Total survey two (n = 137)**	
	**n**	***%***	**n**	***%***	**n**	***%***	**n**	***%***	**n**	***%***	**n**	**%**
On-call (uncertain, unpredictable)	14	*88*	68	*62*	82	65	17	*85*	61	*52*	78	57
Impact on personal life (social, family, work/life balance)	2	*13*	28	*25*	31	25	3	*15*	18	*15*	21	15
Demanding role (adjustment, exhausting, stressful, hard to switch off, takes commitment)	6	*38*	15	*14*	21	17	1	*5*	12	*10*	13	9
Lack of support and respect	2	*12*	17	*15*	19	15	3	*15*	13	*11*	17	12
Burnout	-	*-*	18	*16*	18	14	-	*-*	4	*3*	4	3
Challenges of relationships with women (demands, personality conflict)	-		14	*13*	14	11	1	*5*	4	*3*	5	4
Long hours	4	*25*	10	*9*	14	11	4	*20*	18	*15*	22	16
Isolation	1	*6*	9	*8*	10	8	3	*15*	11	*9*	14	10
Being pioneers (establishing the model, being under scrutiny, implementation)	2	*13*	6	*5*	8	6	-	*-*	-	*-*	**-**	**-**
Issues with remuneration/annualised salary	1	*6*	5	*5*	6	5	2	*10*	5	*4*	7	5
Skills and knowledge required	1	*6*	3	*3*	4	3	1	*5*	2	*2*	3	2
Higher workload in caseload	-	*-*	4	*4*	4	3	-	*-*	5	*4*	5	4
Increased workload for other midwives (including providing care for caseload women)	-		3	*3*	3	2	-	*-*	19	*16*	19	14
Issues with team work	-	*-*	3	*3*	3	2	-	*-*	2	*2*	2	1
Constraints within the hospital (space, rules)	2	*13*	1	*1*	3	2	2	*10*	1	*1*	3	2
Leave not replaced (sick leave, annual leave)	1	*6*	1	*1*	2	2	-	*-*	6	*5*	6	4
Being unavailable for women	*-*	-	*1*	1	1	1	*2*	10	12	10	14	10

There was greater diversity in the responses provided by midwives in standard care. *The impact on midwives’ personal lives* was mentioned by 25% of standard care midwives at baseline, and 15% after two years. *Burnout* (which was not mentioned by any of the caseload midwives) was reported as a negative by standard care midwives in both surveys, although there was a marked decrease between surveys (14% to 3%), suggesting that over time there was less concern that caseload midwifery had the potential to be associated with burnout in these models. *Increased workload for midwives in standard care*, which included comments about taking over care of caseload women increased in frequency between surveys (2% to 14%), suggesting that this was a negative aspect of caseload midwifery that was not anticipated by the midwives in standard care prior to the commencement of the model. *Challenges of relationships with women (including personality conflict)* were also identified by midwives in standard care, but the frequency of this response decreased over time (from 13% to 4%).

Twenty caseload midwives commented on aspects of caseload midwifery that they found to be negative for them personally (Table 8). The most frequently mentioned negative aspect of caseload midwifery for the midwives personally was *on-call work,* which was also the most frequently mentioned in the more generic question of negatives of caseload midwifery for midwives; more than half (12/20) reported this as a negative aspect of the role for them personally. Four caseload midwives identified *long hours associated with peak periods of activity* as a negative aspect for midwives generally, but seven identified this factor as a negative aspect of the role for them personally. A number of single comments reflected some issues not previously identified, such as the *challenges of planning annual leave so far in advance*, *learning how to manage ‘downtime’* and the *difficulties in changing caseload partners*.

**Table 8:** **Negative aspects of caseload identified by caseload midwives for themselves personally (two year survey only)**

***Theme***	***(n = 20)***
On-call (uncertainty, unpredictable)	*12*
Long hours	*7*
Limitations of the model (being unavailable for women)	*3*
Isolation	*3*
Demanding (hard to switch off)	*2*
Impact on plans for leave and travel	*2*
Periods of downtime	*1*
Part-time hours excessive	*1*
Providing back up for others midwives	*1*
Change in partner	*1*
Lack of support (management, staff, Drs)	*1*
Finding balance	*1*

### Resigning midwives Copenhagen Burnout Inventory and Midwifery Process Questionnaire scores

Four midwives who resigned from the caseload model during the study period completed a survey at the time of their resignation; three of these midwives also completed a baseline survey. CBI and MPQ scores were calculated for the resigning midwives. While there does not appear to be any pattern to suggest any association between the MPQ or CBI results and resignation, the small numbers are a limitation on the usefulness of these findings.

## Discussion

This study compared caseload and standard care midwives’ attitudes towards their professional role and their experience of personal, work-related and client-related burnout at two time points; at the commencement of newly introduced caseload midwifery models, and after the model had been operating for two years. Compared with midwives working in standard care models, caseload midwives had more positive views of their professional role and had lower burnout scores.

Two years after the introduction of the new model, caseload midwives’ attitudes to their professional role were more positive than midwives working in standard care. Working in caseload midwifery was associated with an improvement in midwives’ views of their professional role, not only when compared to midwives in standard care, but also for most caseload midwives over time.

The responses to open-ended questions in the survey indicated that there were a number of aspects of caseload midwifery that were viewed as positives for midwives generally, and more specifically for those with experience in the caseload model, which may help in explaining the MPQ findings. Flexibility, continuity and relationships with women and job satisfaction were consistently identified by survey respondents as positive aspects of the role.

Increased satisfaction and professional fulfilment for midwives working in caseload models has been previously reported [5-8,38]. Two studies of midwives’ experiences of caseload midwifery have used the MPQ [5,38] and have reported similar findings to this study. Turnbull et al. developed the tool to measure the views of professional satisfaction, professional support, client interaction and professional development for midwives working in a newly established Midwifery Development Unit (MDU) compared to midwives working in the same hospital in standard midwifery roles at the time of implementation of the model and after 15 months of operation. An improvement in ratings of professional satisfaction, support, client interaction and professional development for the midwives who worked in the MDU was reported, and comparison between groups indicated that ratings were significantly higher in terms of positive attitudes towards their professional role for midwives in the caseload model than those in standard care [38], which is very similar to the findings reported in this study. Similarly, Collins and colleagues [5] used the MPQ to examine the views of 15 midwives working in a MGP in Adelaide, Australia and reported improvement in professional satisfaction, professional support and client interaction, although there was no comparison to midwives working in standard care models in this study.

A number of authors who have explored midwives’ views have made suggestions as to the reasons for the increased satisfaction reported for midwives working in continuity models. These include providing continuity of care, forming relationships with women, and having occupational autonomy [8,10,12-14,22,50,51]. Much of the literature about caseload midwifery reflects the findings in this study, with the relationship with women being a positive and valued aspect of the role [10,52]. A recent Australian mixed-methods study of a caseload model for Indigenous Australian women in the Northern Territory reported that caseload midwives felt that they could ‘really make a difference’ to women [15]. Similarly, McCourt and Stevens [53] reported an involvement of ‘self’ in caseload midwives’ work, and through the personal investment by caseload midwives to their role, they came to view midwifery not as something they ‘did’, instead it was ‘who they were’. Encompassed in this was an investment in work, and a sense of reciprocity as caseload midwifery was seen to benefit both women and the midwives themselves [54].

It has been suggested that burnout may be a risk associated with caseload work because of features of the model, such as long hours associated with on-call work [5,10,14,17], which may result difficulty in achieving work-life balance [14,17,19]. While this study identified that aspects of caseload midwifery, such as on-call and working long hours were consistently identified across all respondent groups as negative features of the model, they do not appear to have contributed to higher burnout, as caseload midwives had significantly lower burnout scores across all three burnout subscales compared to their standard care counterparts after caseload had been in place for two years.

Burnout may be lower in models where the level of occupational autonomy and capacity to build relationships with women is high [12,14,21] and both of these factors were identified by the caseload midwives as positive features of the models in this study. Flexibility, improved lifestyle and personal autonomy are all positive features of caseload work that have been reported in the literature [5,12,17,54,55], and these positive aspects of caseload work may help explain the low burnout scores reported for caseload midwives in this study. While there was a reflection in this study of a negative attitude towards on-call work from all respondent groups, flexibility (which is offered by on-call work) was seen as the most positive aspect of the role for those working in the model. It may be that, in combination with personal autonomy which allows midwives to determine work patterns and protected time off, on-call work may offer flexibility that actually facilitate caseload midwives’ achieving a work-life balance [12]. The industrial regulations that are embedded in Australian continuity models are prescriptive on aspects of work practices such as length of time worked and protected time off-call, which could further help explain the findings in this study.

Other strategies have been suggested to reduce the prevalence of burnout such as accessing social and professional support, and working in groups and small teams, which allows for back-up [14,56]. These factors are designed to support caseload midwives to achieve a work-life balance, and may also account for the lower burnout scores and higher professional satisfaction and professional support that caseload midwives reported in this study. So, while there is no evidence in this study of burnout being associated with caseload midwifery, it does highlight a number of features of caseload work that could positively or negatively contribute to midwives’ views and experiences of the model.

While the concepts of burnout and professional satisfaction are not opposites, the experiences of the caseload midwives in this study would suggest that there are both positive and negative aspects to the role. The positive aspects of caseload midwifery (such as forming relationships with women and working with a level of personal and professional autonomy) may offset the negative aspects (such as the requirement to work on-call). Roles for midwives that enable the establishment of meaningful relationships with women may be a key factor in a sustainable midwifery workforce [57,58], and this may explain the higher level of professional satisfaction that was observed in caseload midwives compared to midwives in standard care models in this study. There was also a contrast in the views between caseload and standard care midwives, particularly when identifying positive aspects of the role. For example, the flexibility within the role was seen as positive by the majority of the midwives working in the model, but identified by less than one quarter of standard care respondents in both surveys. Therefore, it is possible that some of the positive aspects of the role are less apparent to those who have not experienced working in this way. That is, midwives with no experience of working in the caseload model may be less likely to understand the positive aspects of the role, which in turn may act as a deterrent in recruitment of midwives to the model. These factors should be considered by organisations considering the introduction and/or the expansion of caseload models.

There are, however, features of the way that caseload work is organised that may deter or constrain some midwives from choosing to work in this way. Qualitative data collected via in-depth interviews with caseload midwives and key stakeholders alongside the survey data reported in this paper will further explain the concerns that were raised throughout the study about features of caseload work that were deterrents for midwives. Longitudinal studies may also be a way to explore recruitment and attrition trends, and how they may be associated with measures such as satisfaction and burnout, thus informing the issue of sustainability of caseload models.

### Strengths and limitations

There are a number of features of this study that address existing gaps in the caseload midwifery evidence. The study was conducted over a two and a half year period at two sites (a metropolitan tertiary hospital, and a regional hospital), which enabled data collection during the initial phases of implementation, and later when the model had been established for two years. The majority of studies that have explored caseload midwifery to date have been constrained by small numbers of midwives and have been conducted at a single time point at a single site.

Existing evidence about caseload midwifery in the Australian context has provided little comparative data between caseload and standard care midwives. Unlike most other studies, a comparison between the midwives in caseload with other midwives in the organisation has been included in this study, and makes a contribution to understanding if there are associations between caseload work and greater levels of burnout or satisfaction.

Small numbers of midwives resigned from the caseload model throughout this study. Studies with larger numbers of caseload midwives (thus more likely to have greater numbers of resigning midwives), may be better equipped to demonstrate patterns or associations between caseload work and attrition from the model.

Different recruitment strategies were used for the two groups in this study, and while it was thought that anonymity would increase survey returns for the standard care midwives, it does not allow for measurement of changes over time for midwives working in standard care models. It is also possible that responders (both caseload and standard care midwives) differ from non-responders in terms of their views and experiences of caseload midwifery. Caseload midwives self-selected into this work and were very supportive and passionate about this model of care, therefore, arguably, they had a vested interest in seeing it succeed, which may have potentially influenced their responses to the surveys. Equally, while the potential sample included all standard care midwives working in the organisations in midwifery roles, less than 50% responded to each survey, and the views of the non-responders are unknown.

The challenges of conducting research into burnout have been acknowledged, and a number of these limitations also apply to this study. Burnout is a complex, multi-factorial, and subjective experience and thus it is difficult to attribute the development of burnout to any one particular cause. The CBI measures the presence of burnout in different aspects of an individual’s life, which has been helpful in considering if there is any association between the new style of work in caseload midwifery and the presence of burnout, particularly in the work-related or client-related domain. While there was no evidence to suggest that over the two year period of this study caseload midwifery was associated with burnout in any of the three domains, the absence of any evidence to support the association between caseload midwifery and burnout in this study cannot eliminate the possibility that it may appear later. There may also be a higher non-response rate amongst individuals who are experiencing higher levels of burnout [59].

## Conclusion

In this study, caseload midwifery was associated with lower burnout scores and higher ratings of attitude to their professional role, including professional satisfaction. Further research should explore factors that influence midwives’ decisions about working in caseload models and consider factors that are influential in organisational sustainability.
